# Identifying car ingress movement strategies before and after total knee replacement

**DOI:** 10.1080/23335432.2020.1716847

**Published:** 2020-01-22

**Authors:** Dimitrios Sokratis Komaris, Cheral Govind, Jon Clarke, Alistair Ewen, Artaban Jeldi, Andrew Murphy, Philip Riches

**Affiliations:** a Department of Biomedical Engineering, University of Strathclyde, Glasgow, Scotland; b Orthopaedic Department, Golden Jubilee National Hospital, Glasgow, Scotland

**Keywords:** Movement strategy identification, motion analysis, total knee arthroplasty, biomechanics, hierarchical clustering, osteoarthritis

## Abstract

**Background**: Post-operative performance of knee bearings is typically assessed in activities of daily living by means of motion capture. Biomechanical studies predominantly explore common tasks such as walking, standing and stair climbing, while overlooking equally demanding activities such as embarking a vehicle. **Aims**: The aim of this work is to evaluate changes in the movement habits of patients after total knee arthroplasty surgery in comparison to healthy age-matched control participants. **Methods**: A mock-up car was fabricated based on the architecture of a common vehicle. Ten control participants and 10 patients with severe osteoarthritis of the knee attended a single- and three-motion capture session(s), respectively. Participants were asked to enter the car and sit comfortably adopting a driving position. Three trials per session were used for the identification of movement strategies by means of hierarchical clustering. Task completion time was also measured. **Results**: Patients’ movement behaviour didn’t change significantly following total knee arthroplasty surgery. Control participants favoured different movement strategies compared to patients post-operatively. Group membership, height and sidedness of the affected joint were found to be non-significant in task completion time. **Conclusion**: This study describes an alternative movement identification technique for the analysis of the ingress movement that may be used to clinically assess knee bearings and aid in movement simulations and vehicle design.

## Introduction

Optoelectronic methods are frequently adopted to monitor the rehabilitation progress of patients after total knee arthroplasty (TKA) by exploring the body’s kinematics and kinetics during a series of assessments resembling activities of daily living (Smith et al. 2006; Yoshida et al. 2008; McClelland et al. 2011). Predominantly, motion analysis studies explore level walking, sit-to-stand-to-sit and stair ascent/descent (Komnik et al. 2015). Infrequently more physically demanding movements such as squatting (McClelland et al. 2009), walking followed by a sidestep (Leffler et al. 2012) and obstacle crossing (Mandeville et al. 2008) are investigated in order to uncover compensation mechanisms that may not be apparent in level walking (McClelland et al. 2009; Komnik et al. 2015). Yet, such tasks hardly resemble a so-called ‘activity of daily living’ of elderly people living with knee joint implants.

Automobile transportation is vital for both commuting and social interactions, and an inseparable part of today’s living requirements. While the interest of the automobile industry in the ergonomical development of vehicles is increasing, biomechanical studies tend to focus on the implications of human motion in vehicle design (Giacomin and Quattrocolo 1997; Andreoni et al. 2002; Lempereur et al. 2005; Reed and Huang 2008; El Menceur et al. 2008; Chateauroux and Wang 2010). Whilst the comfort and safety of elderly passengers are often addressed and suggestions are offered to enhance their convenience (Petzäll 1995), populations with prostheses are frequently excluded (El Menceur et al. 2009). However, older people, predominantly those reporting osteoarthritis (OA) of the lower limbs, often experience significantly more problems than younger adults, when embarking and disembarking of a car (Herriotts 2005). Thus, this study focuses on the functional performance of elderly patients with a TKA of the knee in a demanding, but common daily activity, namely car ingress.

The difficulty of the task in question arises from the architecture of the vehicle. Typically, the configuration of the side sill, roof and steering wheel, hinders the mobility of the passengers. The interaction of a participant with those elements of the vehicle while performing the movement in a motion caption laboratory, is also the root of complications in the kinematic and kinetic analysis of such recordings. Researchers customarily restrict the movements and habits of the studied population in order to facilitate analysis and allow the comparison of the generated measures: that is, fixing the treadmill’s walking speed (Vogt and Banzer 1999), using chairs without armrests (Farquhar et al. 2009; Abujaber et al. 2015) and staircases without bannisters (Catani et al. 2003), dictating the starting position (Spyropoulos et al. 2013; Abujaber et al. 2015), etc. Nonetheless, vehicle ingress strategies have been shown to feature great diversity in how individuals manoeuvre to get into a car (El Menceur et al. 2009). Thus, restraining the interaction of a subject with the elements of the vehicle may ultimately defeat the purpose of the analysis.

As a precursor to a full kinematic and kinetic analysis of car ingress, this paper examines the car ingress task through the identification of movement strategies by means of hierarchical clustering (HC) of kinematic data. How the adopted ingress strategies vary pre-operationally, post-operationally and one-year post-operationally, is also addressed. Alternatively to HC, other techniques such as machine learning have been excessively used in the study of human biomechanics (Komaris et al. 2019). Yet, clustering approaches were favoured in this work since they are fast and easy to implement, and they do not require large datasets to train models and produce results.

Previously, car ingress movement has been investigated through key frame information (Lu et al. 2016) and visual inspection of optoelectronic recordings (Ait El Menceur et al. 2008; Chateauroux and Wang 2010). Building on the work of Park et al. (2005) clustering methods have been used to identify several ingress movement strategies (Lempereur et al. 2005; El Menceur et al. 2009; Komaris et al. 2018). Yet, to the authors’ knowledge, there are no studies employing movement identification techniques exclusively in OA patients prior and after TKA surgery. The proposed procedure may be used to assess the post-operative performance of knee implants and provide insight on the movement habits of patients with knee prostheses, or other knee pathologies, aiding ingress movement simulations and vehicle design.

## Methods

### Participants

This paper reports on a subgroup analysis of the clinical trial titled ‘Clinical Investigation of the Functional Outcomes of High Congruency Versus Low Congruency Knee Bearings’ registered as NCT02422251 at ClinicalTrials.gov.

Patients with end-stage OA who were scheduled to undergo unilateral TKA in the Golden Jubilee National Hospital in Clydebank, Scotland were invited to take part in the study. Patient volunteers were excluded from the study if they were under 35 years of age, had previous hip or knee replacement procedure carried out in the previous 12 months, had previous ankle surgery, or any past neurologic history (e.g. stroke or Charcot–Marie–Tooth disease). Eligible patient volunteers were suitable to receive any of three knee implants: high congruent mobile, high congruent fixed, and low congruent fixed bearing (B Braun Columbus® total knee systems, Melsungen, Germany). Recruited patient participants were treated by four different orthopaedic surgeons. Outcome assessors were double blinded to the knee implant randomised allocation.

Control participants were recruited from community groups and social clubs. To match the age of the recruited patient participants, control volunteers were invited to the study if they were over 60 years of age. Exclusion criteria included previous hip or knee replacement procedures, previous ankle surgeries, or any musculoskeletal, neurological or sensory deficit.

Control and patient volunteers were requested to attend a single- and three-motion capture sessions, respectively. Patient participants’ sessions took place within 4 weeks prior to the operation, 6–10 weeks after the operation, and around 1 year after the operation. Ten control and 10 patient participants were considered for this analysis. Power analysis indicated that 10 subjects per group would allow us to detect a 45% difference in the frequency of movement strategies (power 0.8, p=0.05).

The study has been reviewed and approved by the West of Scotland Research Ethics Committee 5 and the Strathclyde University Ethics committee. All subjects provided written informed consents prior to participation.

### Anthropometric measures

Age, gender, height, weight and affected knee (for patient participants only) were measured and recorded, while body mass index (BMI) was calculated for patient and control participants alike (Table 1). A one-way ANOVA was also used to determine if there is a statistically significant difference in the BMIs between the means of the control and the patient groups.

**Table 1.** Anthropometric measures

Characteristic	Control group (*n* = 10)	Patient group, pre-op session (*n* = 10)
Age (years), mean ± SD	67.5 ± 7.7	67.9 ± 4.8
Gender (n), female/male	5/5	5/5
Height (mm), mean ± SD	1691.5 ± 122.5	1712.5 ± 88.3
Weight (kg), mean ± SD	71.3 ± 17.5	87.6 ± 11.1
Affected knee, left/right	Not applicable	4/6
BMI ($kg/m^{2}$), mean ± SD	**24.7 ± 3.6**	**29.9 ± 3.9**

Data in bold represent statistically significant characteristics between groups.

### Instrumentation

**Figure 1.**
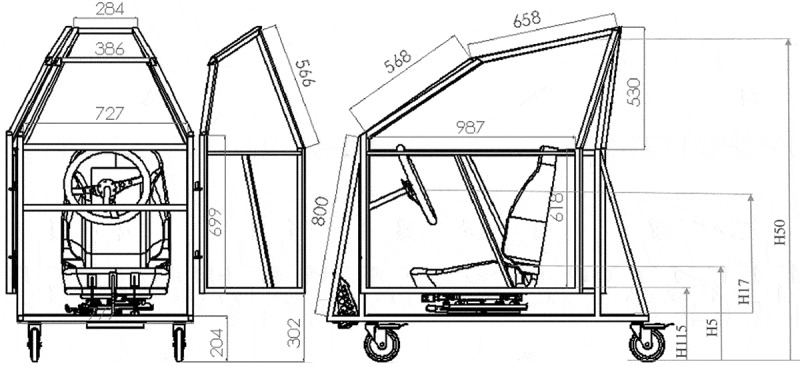
Mock-up car designs; dimensions in mm

**Figure 2.**
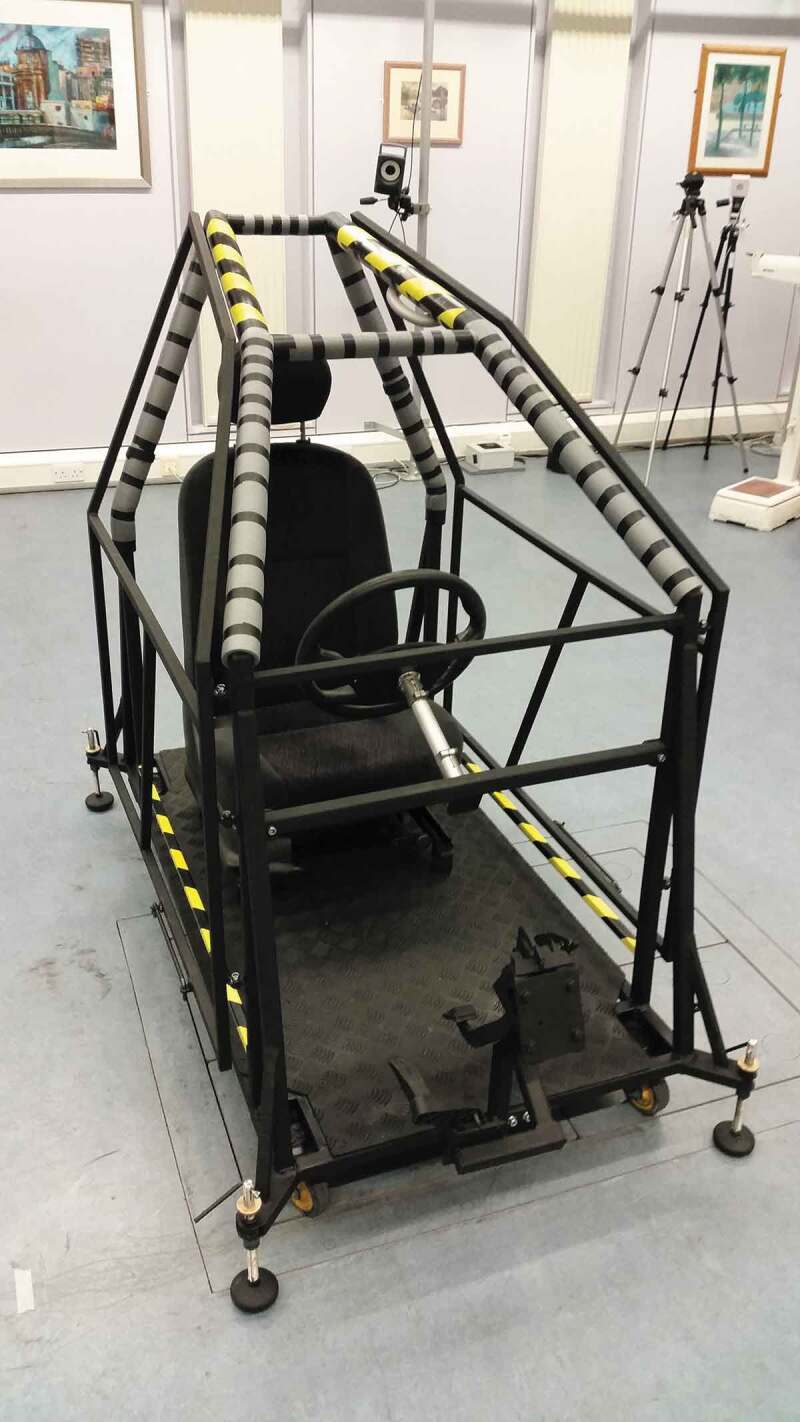
The mock-up car

A right-hand drive mock-up car was designed (Figure 1) and fabricated (Figure 2), with the dimensions based on a Ford Focus hatchback, 1998. According to GOV.UK (2017), Ford is the most popular maker in the UK, accounting for 14% of all cars, while Ford Focus was the second most licensed car at the end of 2017 with 1.3 million vehicles. A driver’s seat, steering wheel, pedals and a roof handle (Ford Focus, 1998) were additionally fitted on the mock-up car. The main dimensions of the doorway (Figure 1, Table 2) are also reported in line with the SAE recommendations (Society of Automotive Engineers 2001).

**Table 2.** Main dimensions of the doorway based on the SAE recommendations

SAE Identifier	Definition	Dimensions (mm)
H5	Seat height^a^ above the ground	490
H17	Steering wheel centre height above the car floor	610
H50	Upper doorway opening to the ground	1200
H115	Sill height above the ground	352

^a^As measured from the rearmost point on the seat.

All measurements were made in a movement Laboratory using a six T-160 and six T-40S camera system (Vicon Motion Systems Ltd, Oxford, UK) at a sampling rate of 100 Hz. Male participants wore tight-fitting Lycra shorts and trainers; female participants additionally wore tight t-shirts. Reflective markers (diameter 14 mm) were affixed onto the skin to thirty-five anatomical landmarks as part of the full-body PlugInGait model (Vicon 2010). A seven-marker subset (seventh cervical vertebra, suprasternal notch, xiphoid process of the sternum, distal fifth metacarpals, and the lateral malleoli) of the full-body model was used for the car ingress strategies identification. The reconstructed full-body model was used for the visual validation of classification results by a single reviewer.

### The movement task

Volunteers were instructed to adjust the seat to their preferable driving position prior to testing. The driver’s door was also adjusted and locked at one of three positions: door fully open at 60°, door partially open at 50°, or at 35°. Participants were then instructed to enter the car, sit comfortably, and place their hands on the steering wheel and feet on the pedals. No other instructions were given. Participants selected their starting position and performed the movement in their own preferred manner. Each participant performed five trials of the ingress movement. The first three successful trials with minimum marker loss were used for the analysis.

### Data analysis

One hundred and twenty trials were initially post-processed using the Vicon Nexus software (Vicon Motion Systems Ltd). Gaps were filled manually in Vicon Nexus with cubic spline interpolations (gaps of seven frames or smaller) and pattern fills. Marker trajectories were filtered using a fourth-order Butterworth filter with a cut-off frequency of 6 Hz. The whole-body centre of mass (COM) trajectory was determined and used to manually isolate two frames, f1 and f2, from each trial. Here, the COM was computed as the weighted sum of all the modelled segments’ center of masses as defined by the full-body PlugInGait marker set. Frame f1 was defined as the initiation of the descending ingress movement as identified by the local maximum of the COM trajectory in the sagittal plane. Frame f2 defined the end of the ingress movement at the local minimum of the abovementioned curve, occurring approximately upon the initial contact of the participant’s buttocks on the driver’s seat.

Global coordinates of the seven-marker subset were exported in ASCII files and used to calculate the following variables from frame f1 to f2: the straight path distance each malleolus marker moved in all global axes normalised by body height; the straight path distance each metacarpal marker moved in all axes normalised by body height; the absolute torso rotation angle about the vertical axis as calculated by the trajectories of the seventh cervical vertebra and suprasternal notch. Subsequently, the variables were organised into three separate matrices corresponding to the feet, hands, and torso movements as follows: a 7×120 matrix containing the progression of the left malleolus marker (columns 2 to 4) followed by the progression of the right (columns 5 to 7); a 4×240 matrix containing the progression of the left metacarpal (rows 1 to 120) followed by the right (rows 121 to 240); a 2×120 matrix containing the torso rotation angles. The first row of each matrix contained a concatenation of a participant identifier (A−J: patient group, K−T: control group), trial number (1−3) and, for the hands matrix, sidedness (LorR). Matrices were submitted to HC (IBM SPSS) separately. Ward’s method and Euclidian distance were the chosen agglomerative algorithm and distance measure, respectively. McNemar and Fisher’s exact tests were then implemented to investigate the differences in the frequency of movement strategies pre and year post-operatively, and between the patient and control groups, respectively.

The time needed to complete the ingress movement, i.e. from frame f1 to  f2, was also measured for each trial. Sets of three trials per participant per visit were averaged to enable comparison among visits, and groups. A repeated mixed measures ANOVA (IBM SPSS) was used to compare the differences in task completion times throughout the patients’ rehabilitation process (pre-, 6 weeks post-, and year post-operative) and due to the sidedness of the patients’ affected joint (left or right knee). A 2×3 ANOVA was also implemented to identify the interaction between control and year post-operative performance, and participants’ height (binned: short, medium, tall) on the task completion time.

### Bespoke questionnaires

Upon task completion, participants were asked to report on (1) the resemblance of the mock-up car to a common car regarding the interior space, legroom, seats and ease of getting in, (2) the resemblance of their movements when performing the car task to those when entering a common/their own car, and (3) whether or not they currently drive a car. Questions 1 and 2 were scaled from 1 (yes, very accurately) to 5 (no, not at all).

## Results

### Anthropometric measures and questionnaires

To detect differences in the BMIs of the two studied groups, a one-way ANOVA was conducted; Shapiro–Wilk test of normality and Levene’s test confirmed that the BMIs were normally distributed for both groups, and that their variances were equal (p > 0.5). There was a statistically significant difference in the BMIs of the control and patient groups (Table 1, p=.006).

Concerning the results of the bespoke questionnaires, participants reported that the mock-up resembled a common car very accurately (85%), to some extent (12.5%), or somewhat (2.5%). When relating the body movements while performing the ingress task to those when accessing a common/their own car, 77.5% described them matching very accurately and 22.5% to some extent. All participants reported as drivers (Table 3).

**Table 3.** Detailed results of the bespoke questionnaires for the patient and control groups

		Results		
Questionnaire		Patients	Controls	All participants
1. Does the mock-up car resemble a common car (interior space, legroom, seats, ease of getting in)?	a. Very accurately	83.3%	90.0%	85.0%
	b. To some extent	13.3%	10.0%	12.5%
	c. Somewhat	3.3%	0.0%	2.5%
	d. Not that much	0.0%	0.0%	0.0%
2. Does this task resemble your movements when entering a common/your car?	a. Very accurately	76.7%	80.0%	77.5%
	b. To some extent	23.3%	20.0%	22.5%
	c. Somewhat	0.0%	0.0%	0.0%
	d. Not that much	0.0%	0.0%	0.0%
3. Do you drive?	a. Yes	100%	100%	100%
	b. No	0.0%	0.0%	0.0%

### Strategies identification

**Figure 3.**
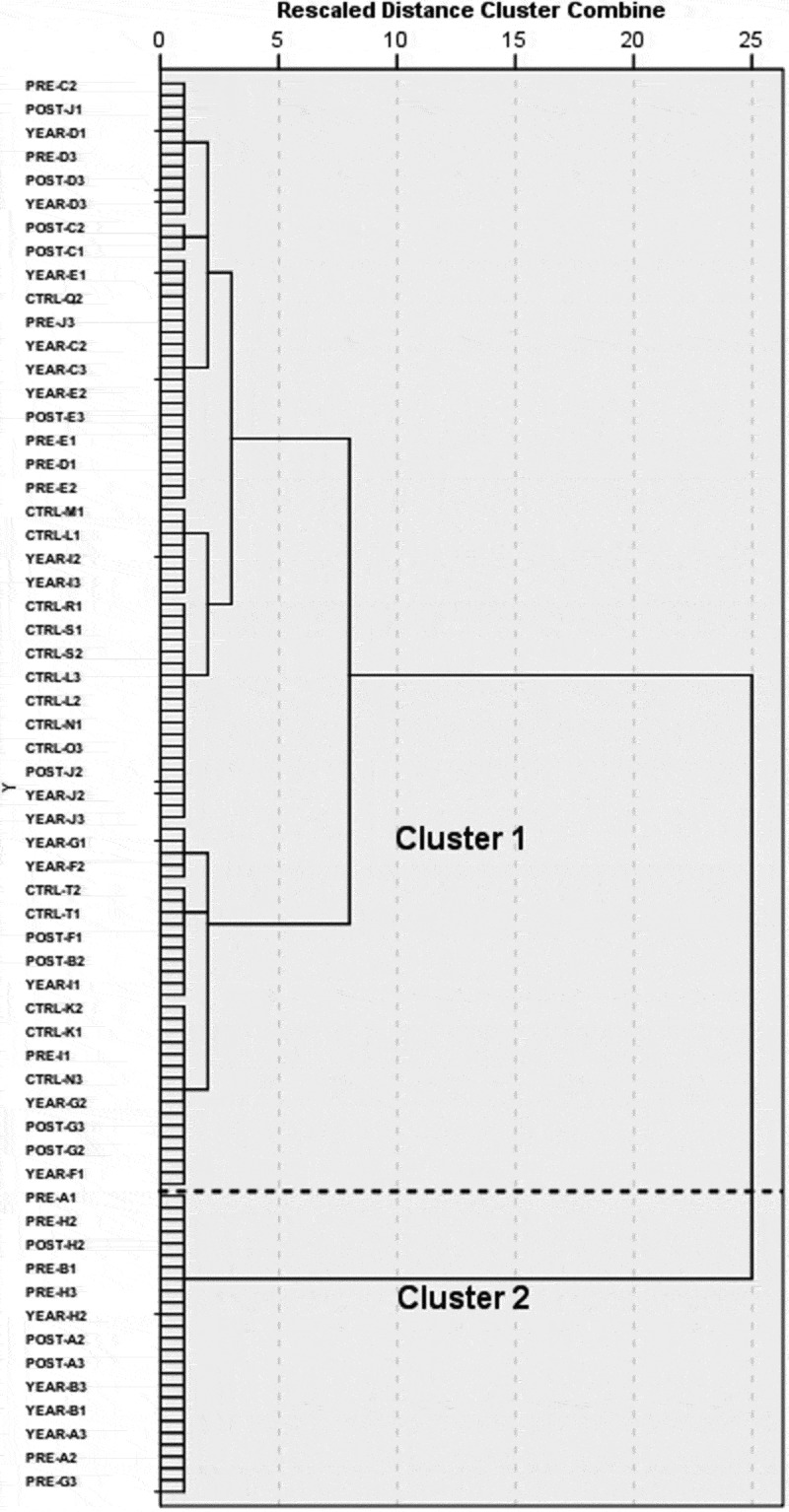
Dendrogram of the HC of the feet movement

The jump in the rescaled agglomeration schedule coefficient from the two- to one-cluster solution (Figure 3), as well as previous numerical and observational studies (El Menceur et al. 2008, 2009) suggest a two-cluster solution for the HC of the feet progression matrix. The two major clusters are separated by a dash line on the dendrogram generated by the clustering procedure (Figure 3). Visual inspection of the trials in Vicon Nexus indicates that trials in cluster 1 and 2 contain participants using the one-foot and two-foot ingress movement strategies, respectively. Specifically, participants adopting the one-foot strategy will initiate the ingress movement with their body parallel to the vehicle’s door, and with the left knee raised and flexed. Then, they will bring their torso inside the mock-up vehicle in a continuous movement, with the left foot landing under the steering wheel and the right still on the ground working as a pivot foot. On the other hand, participants using the two-foot strategy will start the movement with their back turned to the vehicle’s door, and then, sit down while still facing outside the vehicle with both feet on the ground.

**Figure 4.**
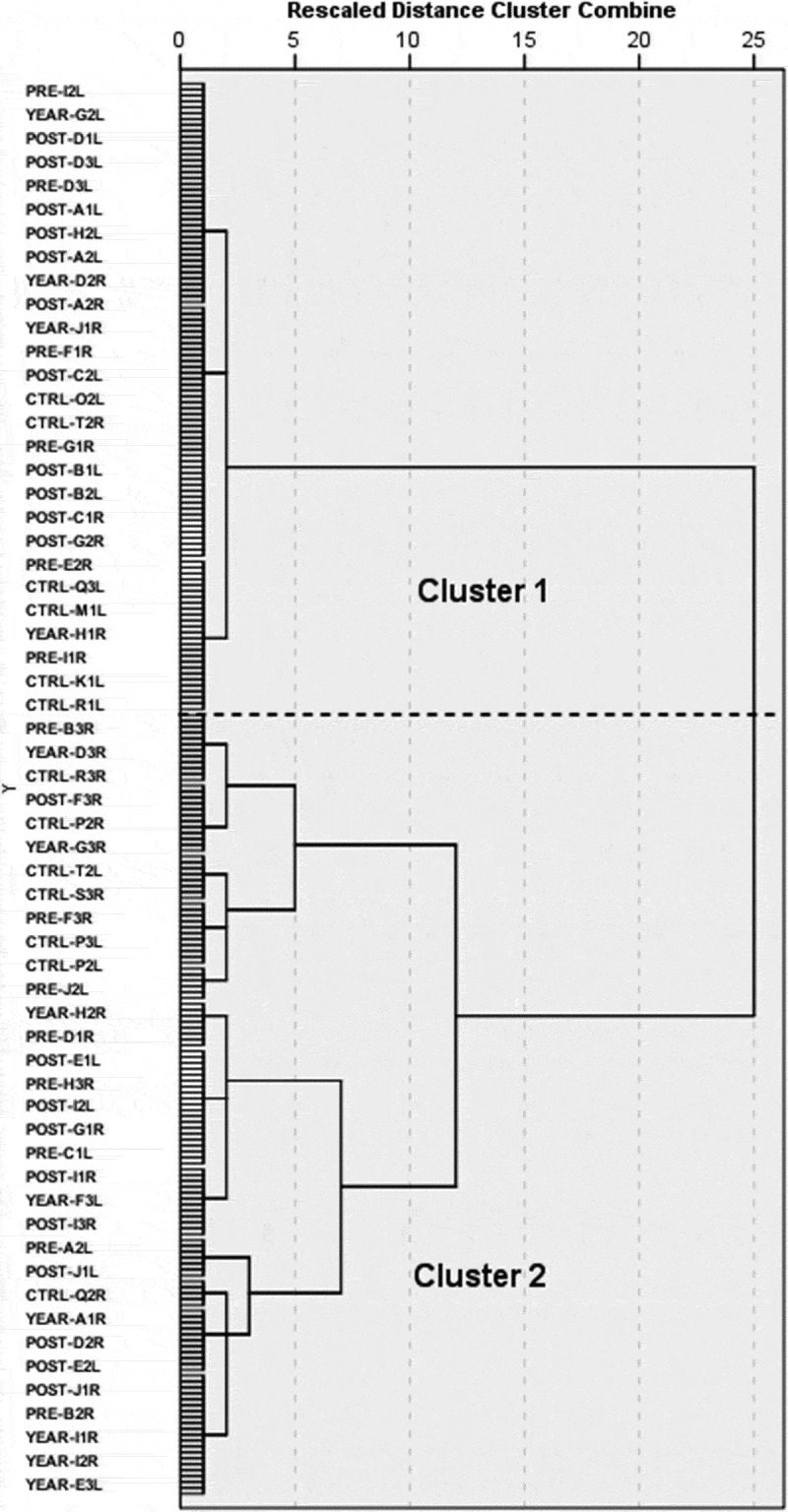
Dendrogram of the HC of the hand movement

Similar to the clustering of the feet movement, the HC of the hand movement separated the elements of the matrix into moving and relatively motionless extremities. Dendrogram (Figure 4) and previous studies (Chateauroux and Wang 2010) confirm the existence of a two-cluster solution. Extremities belonging in cluster 1 and 2 of the hand movement dendrogram, moved on average 45 and 231 mm in space, respectively. Visual inspection of the trials confirmed that the motionless extremities were in fact in contact with an element of the car throughout the majority of the ingress movement. The bilateral behaviour of each participant led to the identification of three strategies describing the hands interaction with the vehicle: no-support, single-support, and double-support. Able-bodied participants adopting the no-support strategy, kept their arms moving freely throughout the ingress movement, and in the majority of the trials, finished the movement with both hands on the steering wheel. Trials of less able participants, clustered in the same category, frequently depict an ongoing attempt to maintain the support of the hands by readjusting their grip on different elements of the environment. Single-support trials portray a pivot hand, typically holding the steering wheel, doorframe, or the seat, whereas the mobile extremity will often swing and grab the wheel by the end of the movement. Finally, double-support trials include participants maintaining support by holding on the steering wheel, door, seat, car frame, or their thighs.

**Figure 5.**
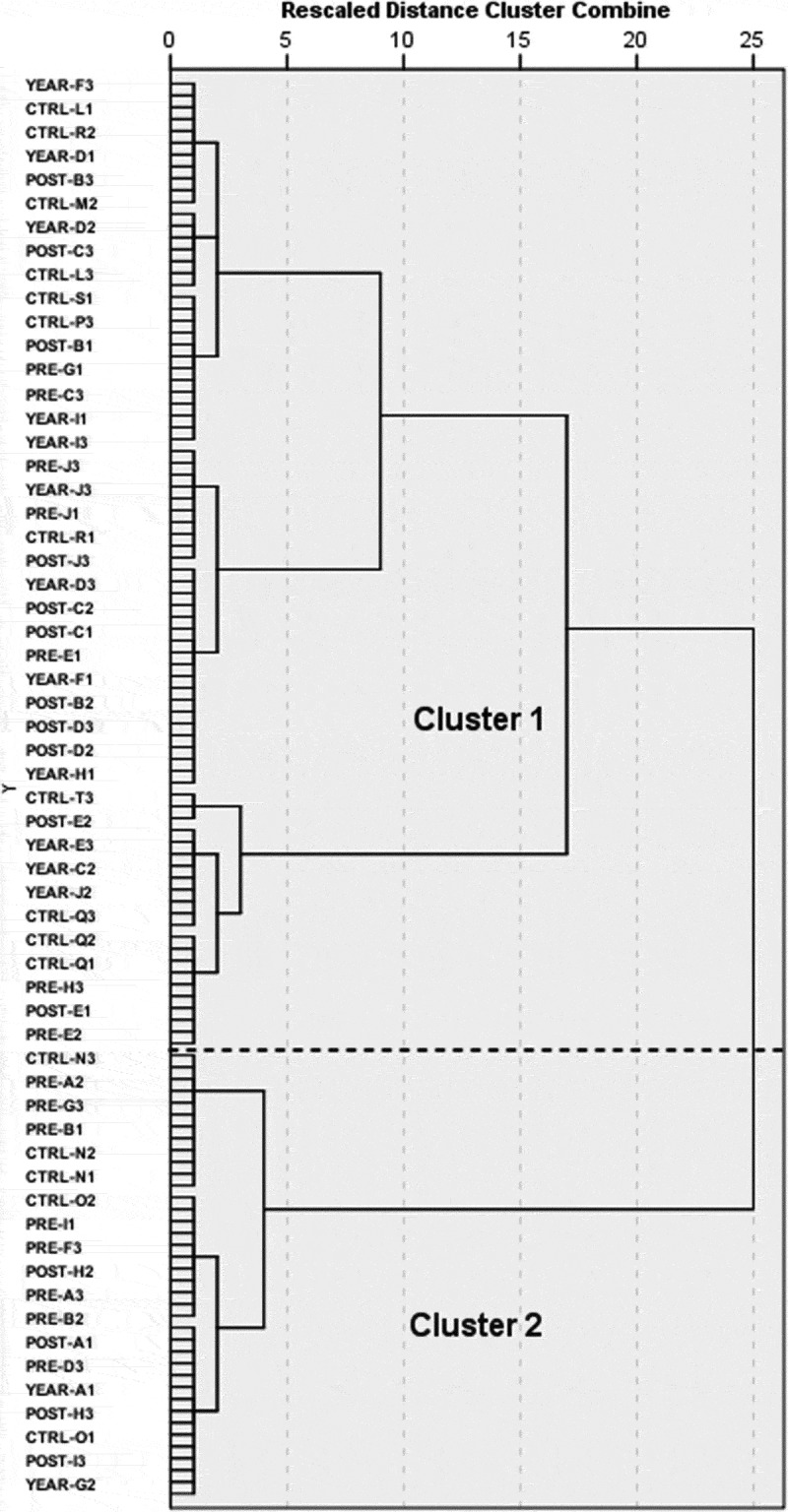
Dendrogram of the HC of the torso rotation

The dendrogram obtained from the HC of the torso rotation matrix suggests a range of solutions, from two to four major clusters (Figure 5); yet, previous research (Lu et al. 2016) proposes allocating the torso movement into two major groups: rotated and straight torso. Trials assigned in the first and second cluster portray participants rotating their torso an average of 32.8° and 6.8° respectively, when entering the vehicle. Participants with increased torso mobility generally tend to rotate their body to face toward the front of the vehicle by the end of their ingress movement. In contrast, participants on the complement cluster will maintain their upper body orientation throughout the task, and in most cases, finish their movement with the steering wheel on their side or back.

The whole-body strategy each participant used to complete the ingress task derives from the accumulation of the three segment strategies identified by the clustering process (Table 4). Apart from the unanimous adoption of the one-foot strategy, control participants favoured the single-support (63%) over the no-support (37%) hand strategy, while the majority of the group (80%) also rotated their torso when entering the vehicle (Table 5). The patient group demonstrated a tendency to switch to the same strategies adopted by control group: 57% and 70% follow the single-support hand strategy pre- and 1 year post-operatively, respectively; 57% and 67% rotated their torso during the same two testing periods; 63% increased to 73% for the one-foot strategy 1 year after surgery. Yet, these changes were found to be negligible: McNemar’s exact tests determined that the performance of the patient group did not improve significantly post-operatively (feet, p=.38; hands: no-support versus single and double support, p=.687; torso, p=.581). Further, Fisher’s exact tests confirmed that the differences in the strategy frequencies between controls and the patients 1-year post-operatively were significant for the feet (p=.005) and hand (p=.01) strategies, but not for the torso (p=.38).

**Table 4.** Strategies distribution

Patient participants				Control participants	
Trial code	First visit	Second visit	Third visit	Trial code	First visit
A1	2F-SS-R	2F-SS-S	1F-SS-S	K1	1F-SS-R
A2	2F-SS-S	2F-DS-S	2F-SS-S	K2	1F-SS-R
A3	2F-SS-S	2F-DS-S	2F-SS-S	K3	1F-SS-R
B1	2F-DS-S	1F-SS-R	2F-DS-R	L1	1F-NS-R
B2	2F-NS-S	1F-SS-R	2F-SS-S	L2	1F-NS-R
B3	1F-NS-R	1F-SS-R	2F-SS-S	L3	1F-NS-R
C1	1F-SS-R	1F-DS-R	1F-SS-R	M1	1F-SS-R
C2	1F-SS-R	1F-DS-R	1F-SS-R	M2	1F-SS-R
C3	1F-DS-R	1F-SS-R	1F-DS-R	M3	1F-NS-R
D1	1F-SS-S	1F-SS-R	1F-SS-R	N1	1F-NS-S
D2	1F-SS-S	1F-SS-R	1F-DS-R	N2	1F-NS-S
D3	1F-SS-S	1F-SS-R	1F-SS-R	N3	1F-SS-S
E1	1F-DS-R	1F-NS-R	1F-SS-R	O1	1F-SS-S
E2	1F-SS-R	1F-NS-R	1F-SS-R	O2	1F-SS-S
E3	1F-SS-R	1F-NS-R	1F-NS-R	O3	1F-SS-S
F1	1F-SS-S	1F-NS-R	1F-NS-R	P1	1F-NS-R
F2	1F-SS-S	1F-NS-R	1F-SS-S	P2	1F-NS-R
F3	1F-NS-S	1F-NS-R	1F-NS-R	P3	1F-NS-R
G1	2F-DS-R	2F-SS-S	1F-SS-S	Q1	1F-NS-R
G2	2F-DS-R	1F-DS-S	1F-SS-S	Q2	1F-SS-R
G3	2F-DS-S	1F-DS-R	1F-SS-S	Q3	1F-SS-R
H1	2F-SS-R	2F-SS-R	2F-DS-R	R1	1F-SS-R
H2	2F-SS-R	2F-SS-S	2F-SS-R	R2	1F-SS-R
H3	2F-SS-R	2F-SS-S	2F-SS-S	R3	1F-NS-R
I1	1F-DS-S	1F-SS-S	1F-SS-R	S1	1F-SS-R
I2	1F-SS-S	1F-NS-R	1F-SS-R	S2	1F-SS-R
I3	1F-SS-R	1F-SS-S	1F-DS-R	S3	1F-SS-R
J1	1F-NS-R	1F-NS-R	1F-SS-R	T1	1F-SS-R
J2	1F-NS-R	1F-NS-R	1F-NS-R	T2	1F-SS-R
J3	1F-NS-R	1F-NS-R	1F-SS-R	T3	1F-SS-R

Foot strategies: one-foot (1F) and two-foot (2F); hand strategies: no-support (NS), single-support (SS) and double-support (DS); torso strategies: straight (S) and rotated (R).

**Table 5.** Car ingress strategies frequencies

		Patient group			
Strategy	Control group (*n* = 10)	Pre-op (*n* = 10)	Weeks post-op (*n* = 10)	Year post-op (*n* = 10)	Total (*N* = 40)
Feet					
One-foot, n trials (%)	**30 (100)**	19 (63)	23 (77)	**22 (73)**	91 (78)
Two-foot, n trials (%)	**0**	11 (37)	7 (23)	**8 (27)**	26 (22)
Hands					
No-support, n trials (%)	**11 (37)**	6 (20)	10 (33)	**4 (13)**	31 (26)
Single-support, n trials (%)	**19 (63)**	17 (57)	14 (47)	**21 (70)**	71 (59)
Double-support, n trials (%)	**0**	7 (23)	6 (20)	**5 (17)**	18 (15)
Torso					
Rotated, n trials (%)	24 (80)	17 (57)	21 (70)	20 (67)	82 (68)
Straight, n trials (%)	6 (20)	13 (43)	9 (30)	10 (33)	38 (32)

Data in bolt represent statistically significant differences between groups. Strategy frequencies between controls and patients one-year post-operatively were significant for the feet (*p* = .005) and hands (*p* = .01), but not for the torso (*p* = .38).

A mixed between-within subjects ANOVA was also conducted with the TKA group to compare the effect of sidedness of the affected joint (left or right knee) in the time needed to complete the task across the rehabilitation process (pre-, 6 weeks post-, and 1 year post-operative). There was no significant interaction between sidedness and rehabilitation stage (p=.13). Moreover, there was no significant main effect for the rehabilitation stage (p=.19), or the sidedness (p=.12). In addition, the two-way ANOVA examined the effect of height, for the control and (one-year post-operative) patient groups, in the time outcome measure. There was no significant interaction between the two variables,p=.21. Furthermore, the main effect of group was non-significant (p=.69), as was the main effect of height (p=.89). Shapiro-Wilk tests of residuals and Levene’s test for homogeneity were carried out for both ANOVAs and the assumptions were met (p > 0.5). All effects are reported as non-significant at p > .05. Mean and standard deviation values for task completion time were also calculated for the above-mentioned sub-groups of the sample (Table 6).

**Table 6.** Task completion times

	Control group (*n* = 30)			Patient group							
				Pre-op (*n* = 30)		Weeks post-op (*n* = 30)		Year post-op (*n* = 30)			
Sidedness				Left	Right	Left	Right	Left		Right	
Time (sec), mean ± SD	--			1.54 ± .42	1.59 ± .29	1.94 ± .37	1.42 ± .40	1.45 ± .04		1.20 ± .22	
**Height**	**Tall**	**Med.**	**Short**					**Tall**	**Med.**		**Short**
Time (sec), mean ± SD	1.17 ± .01	1.55 ± .30	1.39 ± .15	--		--		1.44 ± .05	1.21 ± .22		1.30 ± .25
**Average**											
Time (sec), mean ± SD	1.42 ± .23			1.57 ± .36		1.63 ± .46		1.30 ± .21			

The main effects of rehabilitation stage (*p* = .19) and sidedness (*p* = .12), and the interaction between sidedness and rehabilitation stage (*p* = .13) were statistically non-significant in completion time.

## Discussion

This paper presents a straightforward and rapid procedure to identify and classify different vehicle ingress strategies. A bespoke vehicle was manufactured for the purposes of the study. Participants’ questionnaires verified that the mock-up captured the elements of a real vehicle adequately (Table 3): 85% of all participants reported that the construct resembled an ordinary car very accurately. Yet, designing such an assembly that features all essential components of a real vehicle, while permitting marker tracking, caries certain difficulties. For example, feasible additions such as a handbrake, a gear stick, and a dashboard may significantly improve our design, yet undermining the motion cameras’ line of sight and operation.

The proposed algorithm utilises strategic frames of the captured movement task that enclose the variability of the participant’s movement. In the present study, the COM trajectory was proposed for the key frame identification since a full-body marker model was also adopted for the visual validation of the results; alternatively, indicators such as the trajectory of the pelvis’ markers that give similar patterns to that of the COM (Eames et al. 1999) may be used to assist with frame identification. To quantify the features of the movement, we suggest using the kinematic behaviour of segments’ end-effectors. Additional segments (such as the pelvis or the head) may add to the complexity of the result. Clustering kinematic time series can prove to be puzzling task; in this study, the amplitude of the kinematic curves adequately captured movement features and led to a meaningful HC. Measures of similarity and clustering algorithm descriptions and processes have been addressed in a previous study (Komaris et al. 2018).

The HC process revealed a series of strategies for the lower extremities (one-foot and two-foot), hands (no-, single-, and double-support), and trunk (rotated and straight). We hypothesised that participants adopting the one-foot strategy were more mobile, and capable of comfortably balancing and weight bearing on a single leg. The two-foot strategy, on the other hand, possibly indicated an attempt to protect the affected limb from excessive loading and potential pain or discomfort. Likewise, we assumed that unsupported and single-hand-supported movements were opted from able-bodied participants, while double-supported ingress showed a lack of balance, and an attempt to unload the lower limbs. Nevertheless, this assumption proved to be a generalization: hesitant participants struggling to maintain hand support were occasionally sorted in the unsupported movement cluster. Finally, we speculated that participants showing increased torso mobility optimised their movements in order to lessen the seat positioning phase, and swiftly end their ingress movement in a driving position with their upper body phasing toward the steering wheel. On the other hand, less able-bodied participants would demonstrate a distinct downward ingress movement, followed by the seat positioning phase, where they rotate their pelvis and upper body anti-clockwise.

Patients’ movement preferences were split between all observed strategies with fluctuations in the strategy frequencies throughout the two post-operative visits (Table 5). The performance of the patient group did not vary significantly 1 year post-operatively when compared to the pre-surgery assessment (p>0.05), indicating that there were no changes in movement behaviour even after the restoration of the knee’s mobility and the elevation of pain. In contrast, control participants demonstrated a preference towards the one-foot, single hand support, and rotated torso strategies (Table 5). Patient participants’ 1 year post-operative behaviour was incomparable to the controls’ performance for the foot and hand strategies (p=.01). This possibly indicates that patients continue to adhere to the same movement habits even a year after TKA, while protecting their affected joints and minimising the loading on the knees by adopting the two-foot and hand support strategies.

The observed strategy frequencies in this work conform with analogous findings in the literature; for instance, El Menceur et al. (2008) recruited a mixed population of able-bodied participants of different ages, along with people with hip and knee prostheses, and reported two-foot and one-foot ingress movements with frequencies equal to 21% and 79% of all recorded cases, respectively. These findings are in excellent agreement with metrics reported here: 22% and 78% for the two and one-foot strategies, respectively (Table 5). Likewise, Lu et al. (2016) reported that 81.1% of the studied young individuals rotated their torso when entering the car (by 45° or more), which coincides with the controls’ preferences in our study (80%, Table 5).

Neither the group membership nor the rehabilitation stage were found to be significant in task completion time. Participants’ height and sidedness of the affected joint were also found to be non-significant in the time outcome measure. Although non-significant, the variation in task completion time due to sidedness (Table 6) can be attributed to the functional advantage of a right-side prosthesis when entering a right-hand drive car: adopting the one-foot strategy, allows the participant to keep the operated right limb extended and on the ground, while the non-operated left leg will bear the demands of the task by flexing and adducting.

Even though the architecture of the mock-up car is based on one of the most popular vehicles in the UK, a limitation of the present study arises from the consideration of a single design, which forbids the comparison of the ingress movement in different vehicle types (e.g. SUV). Another limitation ensues from the use of tight t-shirts by the female participants; although it is sensible to provide comfortable clothing to the participating population, markers attached on relatively loose clothing may significantly intervene with the kinematic analysis of the torso and pelvis. Further, the dissimilarity in the BMIs between the control and the patient groups (p=.006) may have also played an influence in movement behaviour, since it is reasonably expected that people with low BMI may access the car with more ease. Finally, the validity of each participant’s chosen strategy may be questioned, since movements were recorded in a laboratory setting, under surveillance, followed by the researchers’ instructions. Yet, based on the questionnaire’s results (Table 3), adopted strategies in this study were considered representative of actual movements in daily living.

The proposed process deals with the processing time problem of motion capture data by demanding merely two frames from each trial for the clustering process. Furthermore, rather than applying the HC with all body segments behaviour simultaneously, this approach suggests considering them individually. By doing so, we were able to dichotomise the sample after each individual HC, identify a series of strategies for the considered body segments, and describe the participants’ behaviour by 12 combinations of whole-body strategies. Decomposing the whole-body behaviour led to identifying strategies independently of the participant performing them (Table 4), while also permitting an easier comparison of the groups in question (Table 5). Concentrating solely on the ingress part of the movement while ignoring the variability of the seat positioning phase limits the range of the classification outcome. Although the seat positioning movements are anticipated to be correlated to the preceding ingress strategy, their analysis may reveal additional insight on the way people with lower limb pathologies perform the task. Even though a limitation of this approach, repeating the procedure for the positioning phase is an option.

In conclusion, we managed to successfully identify and classify human movement behaviour as captured by motion analysis. In addition to the analysis of the car ingress task, the effectiveness of the suggested procedure was previously confirmed in the analysis of sit-to-walk trials (Komaris et al. 2018). The results of the clustering process were used to track the progression of movement patterns throughout the rehabilitation of knee osteoarthritis, in an activity of daily living. Neither participant height nor sidedness of the affected knee joint had an impact on the time needed to complete the assessment. The post-operative movement behaviour of the patients did not change significantly when compared to their pre-operative visits. Additionally, patient participants favoured different movement strategies when compared to controls, indicating that the OA group did not switch to more mobile and dynamic movements after TKA surgery.
